# Deciphering Colorectal Cancer–Hepatocyte Interactions: A Multiomics Platform for Interrogation of Metabolic Crosstalk in the Liver–Tumor Microenvironment

**DOI:** 10.3390/ijms26051976

**Published:** 2025-02-25

**Authors:** Alisa B. Nelson, Lyndsay E. Reese, Elizabeth Rono, Eric D. Queathem, Yinjie Qiu, Braedan M. McCluskey, Alexandra Crampton, Eric Conniff, Katherine Cummins, Ella Boytim, Senali Dansou, Justin Hwang, Sandra E. Safo, Patrycja Puchalska, David K. Wood, Kathryn L. Schwertfeger, Peter A. Crawford

**Affiliations:** 1 Division of Molecular Medicine, Department of Medicine, University of Minnesota, Minneapolis, MN 55455, USAqueat002@umn.edu (E.D.Q.);; 2 Department of Laboratory Medicine and Pathology, University of Minnesota, Minneapolis, MN 55455, USA; reese216@umn.edu (L.E.R.);; 3 Department of Biomedical Engineering, University of Minnesota, Minneapolis, MN 55455, USA; ronox004@umn.edu (E.R.); danso020@umn.edu (S.D.);; 4 Department of Biochemistry, Molecular Biology, and Biophysics, University of Minnesota, Minneapolis, MN 55455, USA; 5 Department of Integrative Biology and Physiology, University of Minnesota Medical School, Minneapolis, MN 55455, USA; 6 Minnesota Supercomputing Institute, Minneapolis, MN 55455, USA; 7 Masonic Cancer Center, University of Minnesota, Minneapolis, MN 55455, USA; 8 Division of Biostatistics and Health Data Science, University of Minnesota, Minneapolis, MN 55455, USA

**Keywords:** cancer metabolism, metabolomics, multiomics, tumor microenvironment

## Abstract

Metabolic reprogramming is a hallmark of cancer, enabling tumor cells to adapt to and exploit their microenvironment for sustained growth. The liver is a common site of metastasis, but the interactions between tumor cells and hepatocytes remain poorly understood. In the context of liver metastasis, these interactions play a crucial role in promoting tumor survival and progression. This study leverages multiomics coverage of the microenvironment via liquid chromatography and high-resolution, high-mass-accuracy mass spectrometry-based untargeted metabolomics, ^13^C-stable isotope tracing, and RNA sequencing to uncover the metabolic impact of co-localized primary hepatocytes and a colon adenocarcinoma cell line, SW480, using a 2D co-culture model. Metabolic profiling revealed disrupted Warburg metabolism with an 80% decrease in glucose consumption and 94% decrease in lactate production by hepatocyte–SW480 co-cultures relative to SW480 control cultures. Decreased glucose consumption was coupled with alterations in glutamine and ketone body metabolism, suggesting a possible fuel switch upon co-culturing. Further, integrated multiomics analysis indicates that disruptions in metabolic pathways, including nucleoside biosynthesis, amino acids, and TCA cycle, correlate with altered SW480 transcriptional profiles and highlight the importance of redox homeostasis in tumor adaptation. Finally, these findings were replicated in three-dimensional microtissue organoids. Taken together, these studies support a bioinformatic approach to study metabolic crosstalk and discovery of potential therapeutic targets in preclinical models of the tumor microenvironment.

## 1. Introduction

The liver is the site of one of six cancers with increasing incidence of primary tumors —hepatocellular carcinoma (hepatoma) [1]. Moreover, the liver is a common site of solid tumor cell metastasis, including breast and colorectal cancers, causing significant morbidity and mortality [2]. Tumor cells within the liver interact with liver resident cell types, including hepatocytes. Metabolic adaptation is essential for tumor success throughout cancer cell transformation, proliferation, and metastasis [3,4]. Cancer cells reprogram their metabolism to meet increased demands for energy, biosynthesis, and redox homeostasis. Studies show this adaptation extends to the tumor microenvironment, where cancer cells can tune and exploit their environment to meet metabolic needs [5,6,7,8]. Within the liver, hepatocytes engage dynamic metabolic programs to support local and systemic physiology. Exploitation of this rich metabolic environment may represent an essential interaction that facilitates tumor cell colonization in the liver niche. However, our understanding of the specific interactions between cancer cells and hepatocytes that drive survival and proliferation in the metastatic liver niche remains limited. Previous studies profiling the metabolism of HCC indicate that transformed hepatocytes engage in aerobic glycolysis and altered lipid and amino acid metabolism [9,10]. Moreover, substrate fuels are used to program neighboring immune cells for repressed anti-tumor responses [7]. Metastasizing cells have been shown to require adaptations that help them overcome the hypoxic liver microenvironment [11,12,13]. But these studies do not consider the role of co-localized, non-transformed hepatocytes. Additionally, these studies are limited in their coverage of the omics landscape.

Metabolomics technologies are well-positioned to reveal potential metabolic adaptation in cancer. Mass spectrometry-based untargeted metabolomics surveys global metabolic shifts among samples by measuring the fluctuations of multiple chemical feature abundances detected as mass-to-charge (*m*/*z*) signal and retention time pairs [14,15,16]. An additional dimension of information can be gained by the convergent use of ^13^C-labeled stable isotope tracers. While static measurement of metabolites provides a snapshot of perturbed influxes or effluxes that lead toward or away from measured metabolites, these measurements often fail to reveal nodes through which shifts occur without significant fold changes in the static abundance of metabolites [17]. Merging the advantages of high-resolution mass spectrometry-based untargeted metabolomics with ^13^C-stable isotope labeling known as isotope tracing untargeted metabolomics (ITUM) provides a unique opportunity to discover dynamic and potentially crucial metabolic pathways [18,19,20,21]. However, studying metabolic communication in the microenvironment is an ongoing challenge [22]. Stable isotopes present an advantage, but developing model systems amenable to ITUM approaches while maintaining physiological relevance is difficult. Here, we present an approach using our untargeted metabolomics and ITUM pipelines in an engineered in vitro model to discover metabolic interactions between colon adenocarcinoma cells (SW480 cell line) and primary hepatocytes. To adapt our pipeline to a mixed cell model, we quantify extracellular and intracellular metabolite pools upon co-culture to identify impacted metabolic pathways. Additionally, we hypothesized that the combined study of differential glucose utilization and metabolite–metabolite relationships could reveal tumor cell metabolic adaptation in the hepatocyte–SW480 microenvironment. Therefore, we present approaches that extend the application of untargeted metabolomics and ITUM to reveal nodes of adaptation in the tumor–hepatocyte microenvironment. Finally, a multiomics integration with the transcriptome links metabolic adaptations to altered functional programs in tumor cells with co-localized hepatocytes.

## 2. Results

### 2.1. Co-Culture of SW480 Cells with Primary Hepatocytes Reprograms Metabolism

**Figure 1:**
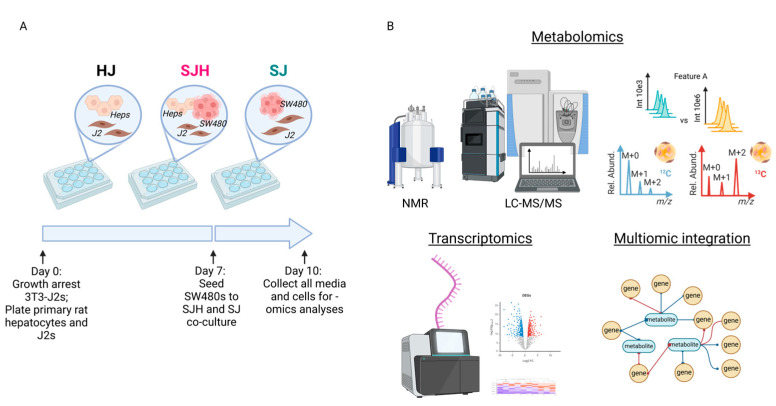
Multiomics study of co-cultures of primary hepatocytes and SW480s. (**A**) Scheme of 2D co-culture system and timeline for cell collection. After growth arrest, 3T3J2s were plated with freshly thawed primary rat hepatocytes for 7 days, and then a subset of these plates received SW480s. After 3 days of co-culturing, all media and cells were collected for analysis. (**B**) Omics coverage of co-cultured groups included broad metabolomic coverage utilizing NMR- and LC-MS/MS-based approaches and bulk RNA sequencing. These data were integrated using univariate approaches to interrogate the impact of metabolomic changes on tumor transcriptional profiles. Abbreviations: HJ, hepatocyte+3T3-J2 co-culture; SJH, SW480+3T3-J2+hepatocyte co-culture; SJ, SW480+3T3-J2 co-culture; NMR, nuclear magnetic resonance; LC-MS/MS, liquid chromatography hyphenated with tandem mass spectrometry.

We used a co-culture model to study the metabolic adaptation of tumor cells to the hepatocyte microenvironment. Culturing primary hepatocytes is challenged by their loss of hepatocyte-like function through de-differentiation [23,24]. However, co-culture with 3T3-J2 murine embryonic fibroblasts can sustain hepatocellular function for more than 6 weeks [25]. Therefore, we directly co-cultured primary rat hepatocytes; murine 3T3-J2 fibroblasts (J2s); and the human colon adenocarcinoma cell line, SW480 (Figure 1A). To form 2D co-cultures, J2s were growth arrested and then plated with hepatocytes in 12-well plates to sustain hepatocellular function (HJ cultures). Control plates of J2s were plated on the same day in preparation for SW480-J2 (SJ) control co-cultures. After 7 days, SW480s were seeded to form SW480-J2–hepatocyte (SJH) co-cultures and SJ controls. All media and cells were collected on day 10 for metabolomics and transcriptomics analyses (Figure 1B). In each experiment, the group of interest, SJH, was compared to HJ and SJ controls.

**Figure 2:**
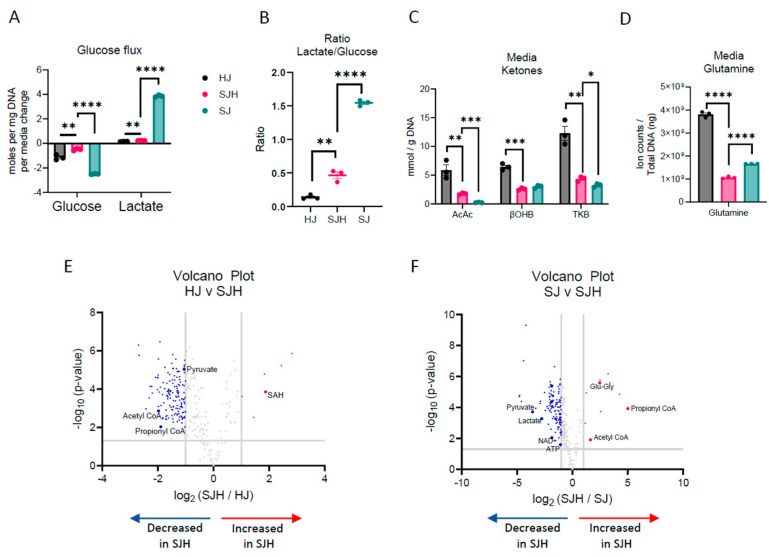
Fuel utilization in 2-dimensional co-cultures. (**A**) Glucose consumption and lactate production in moles per ng total DNA per day. (**B**) Lactate/glucose ratio of media concentration after 24 h. (**C**) Concentration of acetoacetate (AcAc), β-hydroxybutryrate (βOHB) and total ketone bodies (TKB) in mmol/g DNA measured in media after 24 h of incubation. (**D**) Relative abundance of glutamine in total ion counts after normalization to total ng DNA. Volcano plot showing upregulated and downregulated metabolites in SJH compared to (**E**) HJ control cultures and (**F**) SJ control cultures; positive log2FC = up in SJH. Significance tested using unpaired *t*-test, comparison HJ vs. SJH or SJ vs. SJH, and corrected for multiple comparisons using Benjamini–Hochberg method. * *p* adj. < 0.05, ** *p* adj. < 0.01, *** *p* adj. < 0.001, and **** *p* adj. < 0.0001. Abbreviations: HJ, hepatocyte+3T3-J2 co-culture; SJH, SW480+3T3-J2+hepatocyte co-culture; SJ, SW480+3T3-J2 co-culture.

Cancer metabolism is hallmarked by high glucose consumption and utilization through aerobic glycolysis, resulting in high lactate production, known as the Warburg Effect [26,27]. Hepatocytes play significant roles in regulating glucose homeostasis. We hypothesized that hepatocytes may impact glucose metabolism in co-cultures. Therefore, we quantified net changes in glucose and lactate concentrations in conditioned cell culture media using 1H-nuclear magnetic resonance (NMR) spectroscopy. Then, we normalized changes in exogenous metabolite concentrations to cellular biomass (DNA content) to report net glucose consumption and lactate production over 24 h. As expected, SJ controls model the Warburg effect in culture, consuming 2.50 ± 0.01 mol glucose/day/mg DNA, and producing 3.86 ± 0.05 mol lactate/day/mg DNA (Figure 2A). The presence of hepatocytes decreased total glucose consumption by 80% (0.5 ± 0.05 mol glucose/day/mg DNA) and lactate production by 94% to 0.23 ± 0.01 mol lactate/day/mg DNA. Furthermore, the ratio of lactate to glucose significantly decreased from 1.54 ± 0.02 to 0.46 ± 0.05, suggesting that the presence of hepatocytes disrupts Warburg metabolism of SW480s (Figure 2B).

To determine if decreased utilization of glucose by SW480 cells when co-cultured with hepatocytes could result from a fuel switch from glucose to other available substrates, we quantified hepatocyte-derived ketones and culture media-derived glutamine. Using a UHPLC-MS/MS-based approach, we quantified total ketone bodies in the conditioned media of co-cultures after 24 h. As expected, primary rat hepatocyte control co-cultures produced the ketone bodies acetoacetate (AcAc) and β-hydroxybutyrate (βOHB, Figure 2C). In the presence of SW480s, total ketone bodies recovered in the media were diminished by 64%, suggesting either decreased production by hepatocytes or increased consumption of ketones by non-hepatocyte cells. Glutamine abundance was also significantly decreased in SJH co-cultures relative to both HJ and SJ controls (Figure 2D). Interestingly, glutamine abundance decreased 36% in SJH relative to SJ controls, suggesting that the presence of hepatocytes further enhanced glutamine dependence of SW480s. Together, these data suggest an adaptation to fuel utilization in hepatocyte–SW480 co-cultures.

To survey metabolic interaction-dependent metabolite shifts resulting from co-culture, we next used differential analysis of features detected by LC-MS-based label-free untargeted metabolomics. Prior to any downstream analysis, low-variance features were removed; features of interest were annotated in Compound Discoverer 3.3, and when possible, they were matched to commercial standards and MS/MS spectral libraries. Five metabolic features by LC-MS-based untargeted profiling increased more than 2-fold (log2 ≥ 1) in SJH compared to HJ controls. This included putative S-adenosylhomocysteine (SAH; Table 1 and Figure 2E), an intermediate of one-carbon metabolism. Compared to HJ, SJH co-cultures showed 142 downregulated features, including pyruvate (Supplementary Figure S1A), acetyl-CoA (Supplementary Figure S1B), and propionyl-CoA (Supplementary Figure S1C), metabolites important in glucose metabolism and the TCA cycle. Relative to SJ controls, SJH co-cultures show a greater than 2-fold increase in 10 features, including propionyl-CoA (Figure 2F and Supplementary Figure S1C), glutamyl-glycine, an intermediate of glutathione metabolism, and acetyl-CoA (Supplementary Figure S1B). A ≥2-fold decrease was observed in 119 features in SJH compared to SJ controls, including pyruvate (Supplementary Figure S1A), lactate (Supplementary Figure S1D), NAD+ (Supplementary Figure S1E–G), and ATP (Supplementary Figure S1H). However, decreases in the NAD+ pool did not lead to a significant change in the NAD+/NADH ratio in SJH relative to SJ (Supplementary Figure S1G). Finally, we calculated energy charge to determine the current energy status of the co-culture based on relative abundance of AMP, ADP, and ATP pools. We saw a modest, but not statistically significant, decrease in SJH relative to SJ co-cultures, suggesting a possible decrease in available energy (Supplementary Figure S1H,I).

**Figure 3:**
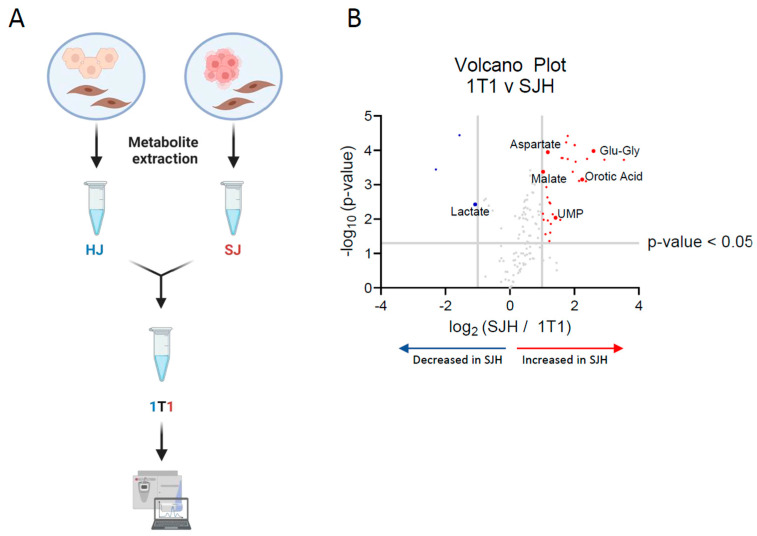
Analytical dilution of co-culture controls reveals metabolic adaptation in SJH co-cultures. (**A**) Schematic of analytical dilution of HJ and SJ controls to form a 1-to-1 ratio (1T1) after metabolite extraction. (**B**) Volcano plot upregulated and downregulated metabolites in SJH compared to 1T1 ion counts; positive log2FC = up in SJH. SJH, SW480+3T3-J2+hepatocyte co-culture; 1T1, 1-to-1 ratio of SJ to HJ metabolite extractant.

Untargeted metabolomics of mixed cell populations represent metabolite pools that are combined across all cell types. Therefore, changes in relative abundance may represent an adaptation in one cell type, a combination of adaptations across multiple cell types, or could simply be a product of dilution of the pool after adding biomass. Due to the observed number of features that were significantly decreased in SJH relative to either HJ or SJ controls, we sought to determine if our approach detects true biological interactions in hepatocyte and SW480 co-cultures, rather than simple dilutions of HJ or SJ metabolite pools. Therefore, we implemented a dilution approach to identify those features that were significantly different from a HJ-SJ dilution (Figure 3A). HJ and SJ extracts were combined 1:1 (1T1) prior to LC-MS injection, alongside HJ, SJ, and SJH samples. Putative metabolites with pool sizes that significantly differed between SJH and controls were compared to the 1T1 dilution sample. A low threshold for discovery was used (*p* < 0.05) to identify those features that may indicate metabolic interactions. Lactate was significantly decreased in SJH relative to 1T1 samples (Figure 3B; Table 2). Normalized ion counts for each group and the analytical control, 1T1, are shown in Supplementary Figure S2, demonstrating a likely metabolic interaction between SW480s and hepatocytes beyond an outcome that could be explained by simple metabolite pool dilution. Additionally, four glutamyl peptides, namely glutamyl-glycine, uracil, uridine diphosphate (UDP), aspartate, and malate, were increased more than two-fold in SJH relative to 1T1 (Figure 3B; Table 2). Employment of an analytical 1T1 dilution of controls for differential analysis of directly co-cultured cells revealed biological derangement of metabolite abundances belonging to glycolytic, amino acid, and nucleoside pathways.

### 2.2. Metabolite Exchange of Nucleoside Intermediates

**Figure 4:**
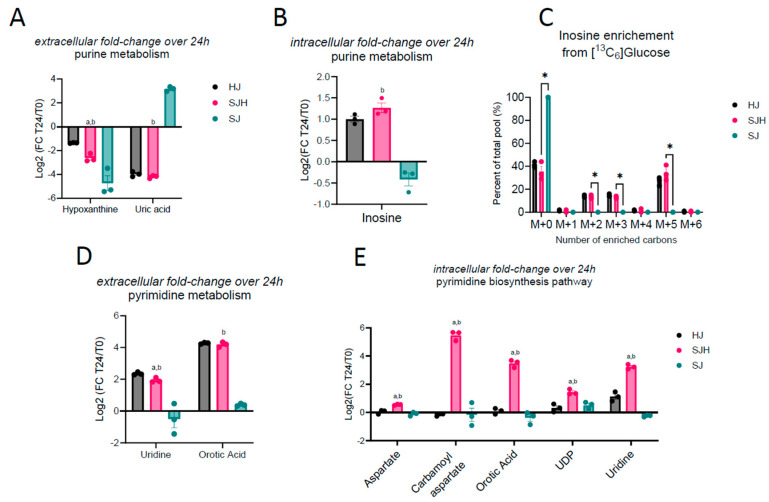
Metabolic interactions of biosynthetic pathways in SJH co-cultures. Fold change of metabolite abundance after 24 h co-culture relative to time point 0 in (**A**) media abundance of purine metabolism products, hypoxanthine and uric acid; (**B**) intracellular inosine pools; (**D**) media abundance of pyrimidine biosynthesis intermediates, uridine and orotic acid; and (**E**) intracellular pyrimidine intermediates and substrates, aspartate, carbamoyl aspartate, orotic acid, UDP, and uridine. (**C**) ^13^C-enrichment of intracellular inosine pools from 22 mM [U-^13^C_6_]glucose in 3D microtissue organoids. Statistical comparison by unpaired *t*-test; letters indicate significance in comparison to HJ controls (“a”) or SJ controls (“b”). * *p* adj. < 0.05. HJ, hepatocyte+3T3-J2 co-culture; SJH, SW480+3T3-J2+hepatocyte co-culture; SJ, SW480+3T3-J2 co-culture; UDP, uridine diphosphate.

Untargeted metabolomics results indicate altered activity in both purine and pyrimidine pathways when SW480s are co-cultured with hepatocytes, whose identities were confirmed by retention time and MS/MS fragmentation match to commercial standards (Figure 2E,F and Figure 3B). We hypothesized that the presence of hepatocytes may facilitate adaptation through exchange of purine and pyrimidine intermediates. Therefore, we performed LC-MS-based untargeted metabolomics on cell extracts and media after the first 24 h of direct co-culture to evaluate fold changes in nucleoside intermediate abundance over time. In all three groups, hypoxanthine, an intermediate of purine metabolism, was depleted comparing starting media (t0) to media harvested after 24 h, indicating uptake from serum-containing media or conversion to other metabolic products (Figure 4A; Table 3). SJH co-cultures had 23% (±3%) greater depletion of hypoxanthine compared to HJ controls and 12% (±4%) less than SJ controls (Supplementary Figure S3).

Interestingly, hypoxanthine abundance was coupled with diminished extracellular uric acid, the terminal product of purine degradation, in HJ and SJH groups, while SJ controls show an average 809% increase in uric acid (Figure 4A and Supplementary Figure S3). Hypoxanthine and uric acid are both metabolites that can be found in starting media due to the presence of serum. We further analyzed media under normal culture conditions and in the absence of cells to control for possible spontaneous degradation and observed an accumulation of hypoxanthine and uric acid (9% and 47%, respectively) after 24 h, suggesting instability of upstream metabolites at 37 °C (denoted as dotted lines; Supplementary Figure S3). Therefore, these data indicate significant metabolic activity in the purine degradation pathway in SJ controls that is altered by the presence of hepatocytes. To investigate if this impacts intracellular purine intermediates, we measured inosine in cellular extracts. Inosine, an intermediate in the purine salvage pathway, showed a 2-fold increase in hepatocyte-containing cultures, while it was modestly diminished in SJ controls after 24 h (Figure 4B). Finally, inosine was uniquely enriched from [U-^13^C_6_]glucose in HJ and SJH groups but not detectable in SJ in 3D microtissue organoids (Figure 4C). Conversely, the average total ^13^C-enrichment of HJ and SJH groups was 58.7% (±1.5%) and 64.7% (±4.5%) of the total inosine pool. This observation suggests that changes in glucose contribution to inosine pools may be localized to hepatocytes. Together, these data suggest the presence of hepatocytes increases purine salvage, rescuing cells from uric acid accumulation.

#### Pyrimidine Metabolism Is Increased in Hepatocytes by Co-Culture with SW480 Cells

Hepatocytes are a primary source of uridine, an intermediate of pyrimidine metabolism. We hypothesized that altered pyrimidine pools may be a result of newly available uridine from hepatocytes. Therefore, we measured pyrimidine intermediates by LC-MS/MS in media and cell extracts to observe changes in pool sizes over 24 h compared to starting media. As expected, we observed an accumulation of uridine in the media of hepatocyte-containing co-cultures that was not present in SJ controls (Figure 4D). Interestingly, we observed an even greater accumulation of orotic acid, an intermediate of de novo pyrimidine biosynthesis (Table 3). We did not observe a significant difference between SJH and HJ controls, suggesting these media signals are primarily driven by hepatocytes. However, intracellular pool sizes of pyrimidine metabolites showed greater increases in SJH relative to HJ controls, including carbamoyl aspartate—the product of the rate-limiting step in de novo pyrimidine biosynthesis—suggesting the co-localization of hepatocytes and SW480s upregulates the pyrimidine biosynthetic pathway (Figure 4E).

### 2.3. Discriminant ^13^C-ITUM Analysis of SJH Co-Cultures

**Figure 5:**
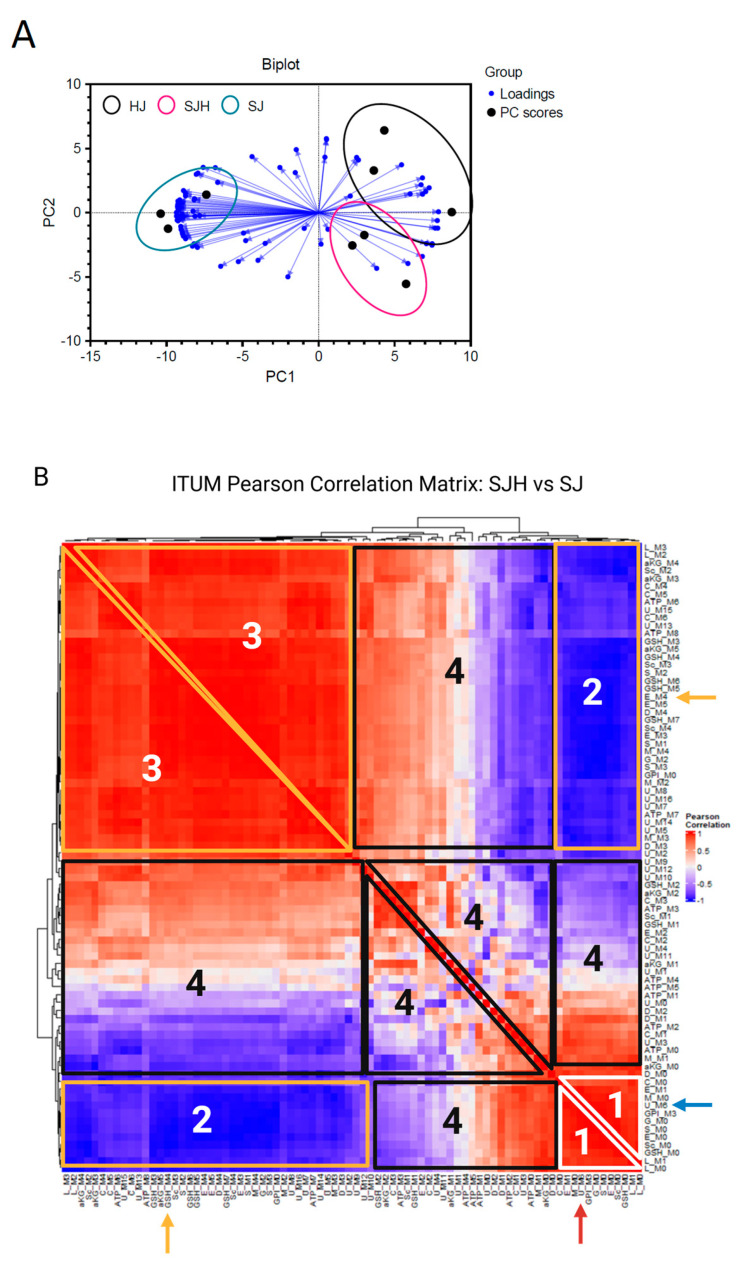
Discriminant ITUM analysis of SJH co-cultures. (**A**) Biplot of first two principal components (PC1 and PC2) of PCA of HJ, SJH, and SJ co-cultures by ^13^C-glucose-enriched isotopologues. Black-filled circles represent samples. Spheres show co-culture groups. Blue directed vectors show isotopologue loadings for PC1 and PC2. (**B**) Hierarchical clustering of ITUM SJH vs. SJ Pearson correlation matrix. White triangle with “1” label indicates control cluster of isotopologues; yellow shapes with “2” and “3” labels correspond to cluster of isotopologues from region of strongly co-enriched isotopologues in response to co-culture; and black shapes with “4” label correspond to cluster of isotopologues with weak co-enrichment in response to co-culture. Red arrow, U_M6 positive correlation with unenriched M+0 isotopologues in Region 1; blue arrow, U_M6 negative correlation with multiple isotopologues, including GSH_M3, in Region 2; and yellow arrow, GSH_M4 positive correlation with metabolic precursor E_M4 in Region 3. Abbreviations: HJ, hepatocyte+3T3-J2 co-culture; SJH, SW480+3T3-J2+hepatocyte co-culture; SJ, SW480+3T3-J2 co-culture; .M#, isotopologue representing number of heavy carbons present in the molecule (i.e., M1 indicates presence of 1 heavy 13-carbon); aKG, alpha-ketoglutarate; S, serine; M, malate; L, lactate; C, citrate; ATP, adenosine triphosphate; U, uridine diphosphate N-acetylglucosamine; G, glycine; D, aspartate; E, glutamate; GSH, glutathione; Sc, succinate; GPI, glycerophosphoinositol.

^13^C-stable isotope tracing of metabolic substrates has been used to detect metabolic adaptation [20,28]. However, incorporation of glucose-derived carbon in diverse metabolic pathways in mammalian cells convolutes biological interpretation of enrichment patterns in complex samples. We hypothesized that informatic integration of co-^13^C-enriched metabolites elucidates pathway activity. Therefore, we employed univariate and multivariate statistical approaches to characterize glucose utilization in co-culture. SJ and SJH co-cultures were treated with [U-^13^C_6_]-glucose for 24 h on day 9. On day 10, cells were snap-frozen for ^13^C-ITUM, and acquired LC-MS data were analyzed for ^13^C-enriched mass isotopomers (i.e., isotopologues) [M + 0, M + 1, …, M + n] to identify nodes of glucose-derived metabolism that differentiate SJH co-cultures from SJ controls. We first used principal components analysis (PCA) to identify isotopologues that discriminate SJH co-cultures from HJ and SJ controls (Figure 5A). PC1 significantly separated SJH and SJ groups, while PC2 moderately separated SJH from HJ controls. Strong association with PC1 indicated co-^13^C-enrichment of these metabolites captures the impact of hepatocytes on SW480s. To further investigate adaptations to glucose metabolism after co-culturing hepatocytes on SW480s, we performed a univariate correlation analysis of top contributors to PC1 loadings, including only SJ and SJH ^13^C-enrichment. We visualized these relationships in a correlation matrix (Figure 5B and Supplementary Figure S4). A positive correlation between feature pairs indicates co-^13^C-enrichment of isotopologue pools in response to SW480 co-culture with hepatocytes relative to SW480 alone, while a negative correlation indicates a possible bifurcation of ^13^C-labeled carbon because of co-culture. As would be expected, fractional enrichments of M+0, the isotopologue indicating no ^13^C incorporation, of several metabolites, including TCA cycle intermediates, were positively associated with each other and clustered together (“Region 1” on Figure 5B). These M+0 species show a strong negative correlation with the ^13^C-enriched isotopologues (species in “Region 2”), e.g., M+0 of glutamate (“E_M0”) versus the incorporation of four ^13^C atoms (“E_M4”). This is the expected relationship and supports the validity of this analytical framework, which also reveals many unanticipated relationships. For example, the M+6 isotopologue of uridine diphosphate N-acetylglucosamine (UDPGlcNAc, corresponding to the direct incorporation of a labeled glucose molecule into glucosamine), clustered with unenriched (M+0) isotopologues of several metabolites in Region 1 (red arrow, Figure 5B) and negatively correlated with enriched glutathione (e.g., GSH_M3, found in Region 2; blue arrow). UDPGlcNAc M+6 enrichment decreases in co-culture, while enrichment of GSH increases (relative to the SJ condition), suggesting glucose is redirected from the hexosamine biosynthetic pathway to glutathione synthesis in co-culture (Supplementary Figure S5). Region 3 shows a cluster of isotopologues from glycolytic and TCA cycle intermediates with strong co-enrichment. As expected, this includes enriched isotopologues of metabolites in the same pathway, such as glutamate (E_M4), a precursor to GSH (GSH_M4; yellow arrows, Figure 5B). The positive co-enrichment of other TCA cycle intermediates with GSH may meet GSH demand in response to co-culture. Finally, Region 4 shows isotopologues with nominal relationships with each other, suggesting little change in response to co-culture. Together, these data indicate that ^13^C-enrichment of glutathione from glucose is significantly impacted by the co-culturing of SW480s and hepatocytes. Glutathione is an important metabolite in redox homeostasis within the cell, and changes to its biosynthesis may be a significant adaptation of metabolic pathways to the tumor–hepatocyte microenvironment.

### 2.4. Transcriptomic Analysis of 3-Dimensional Hepatocyte–SW480 Microtissue Organoids

**Figure 6:**
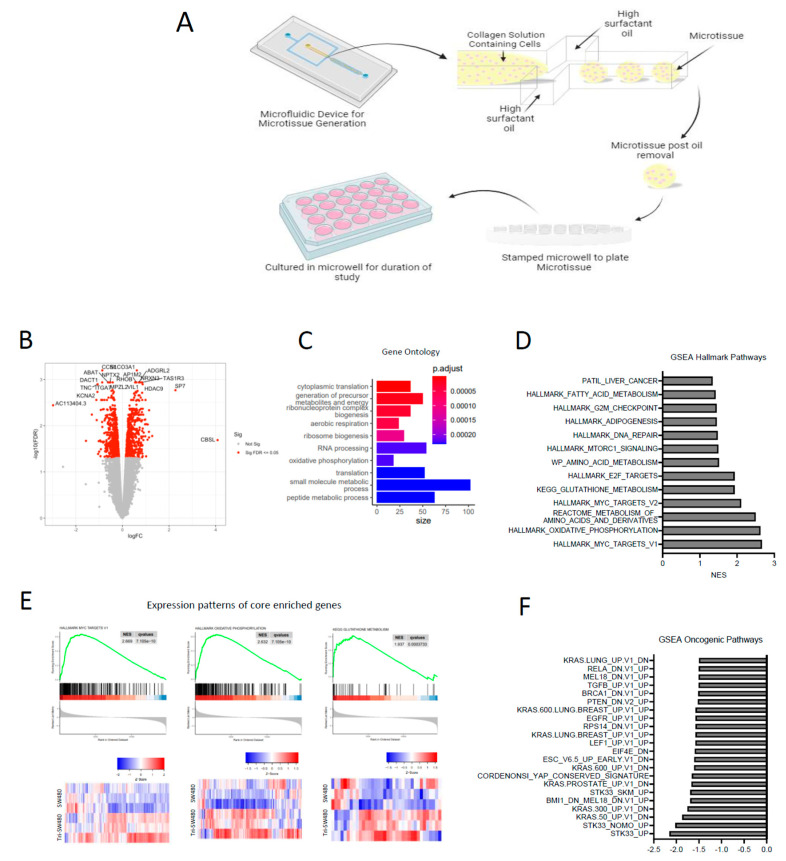
Transcriptional profiling of tumor cells in 3D microtissues identifies alterations in metabolic pathways upon exposure to hepatocytes. (**A**) Three-dimensional microtissue organoid scheme. (**B**) Volcano plot showing significantly upregulated and downregulated genes in samples from SJH cultures compared with SJ cultures. Positive logFC indicates up in SJH cultures. (**C**) Gene ontology analysis using DEG as input. (**D**) GSEA hallmark analysis of SJ compared with SJH. A positive NES score indicates gene profiles that are enriched in tumor cells from the SJH condition compared with the SJ condition. All shown pathways adjusted FDR < 0.05. (**E**) Expression patterns of core enriched genes associated with Myc pathway and two metabolic pathways, oxidative phosphorylation and glutathione metabolism, that are positively enriched in the SJH condition and heat maps. (**F**) GSEA Oncogenic analysis of SJ compared with SJH. A negative NES score indicates gene profiles that are enriched in tumor cells from the SJ condition compared with the SJH condition. All shown pathways adjusted FDR < 0.05. HJ, hepatocyte+3T3-J2 co-culture; SJH, SW480+3T3-J2+hepatocyte co-culture; SJ, SW480+3T3-J2 co-culture.

To evaluate SW480 adaptation to co-culturing with hepatocytes in a more physiological setting, we also performed transcriptomic profiling of SJ and SJH groups using a 3D microtissue organoid model. We co-cultured SW480 with primary rat hepatocytes and 3T3-J2 fibroblasts within collagen I-based microtissues (Figure 6A). Conditions included SJ cells and SJH using the same cell numbers and proportions as 2D co-culture. The SJH tricultures alongside co-culture controls were maintained for 7 days before harvesting for transcriptional and metabolic analyses. To identify transcriptional alterations that occur in cancer cells upon exposure to hepatocytes, we performed bead isolation using antibodies against CD326 (EpCAM) to isolate the SW480 tumor cells grown in the presence and absence of hepatocytes and supporting J2s, and we performed bulk RNA-seq analysis to identify genes and pathways that are modulated when exposed to hepatocytes. We found that exposure of tumor cells to hepatocytes led to increased expression of 708 genes and decreased expression of 762 genes in SW480 cells (adj. *p* < 0.05) (Figure 6B). Further analysis of gene ontology (GO) and gene set enrichment analysis (GSEA) demonstrated significant alterations in hallmark pathways of Myc targets and pathways associated with several metabolic processes, including oxidative phosphorylation, metabolism of amino acids, GSH metabolism, and fatty acid metabolism (Figure 6C–E; Supplementary Tables S1 and S2). We also compared RNAseq findings to oncogenic pathways (Figure 6F). These findings demonstrate that hepatocytes drive transcriptional changes in tumor cells, many of which are associated with changes in metabolic pathways.

### 2.5. Multiomics Analysis of Differentially Expressed Genes and Static Metabolite Pools

**Figure 7:**
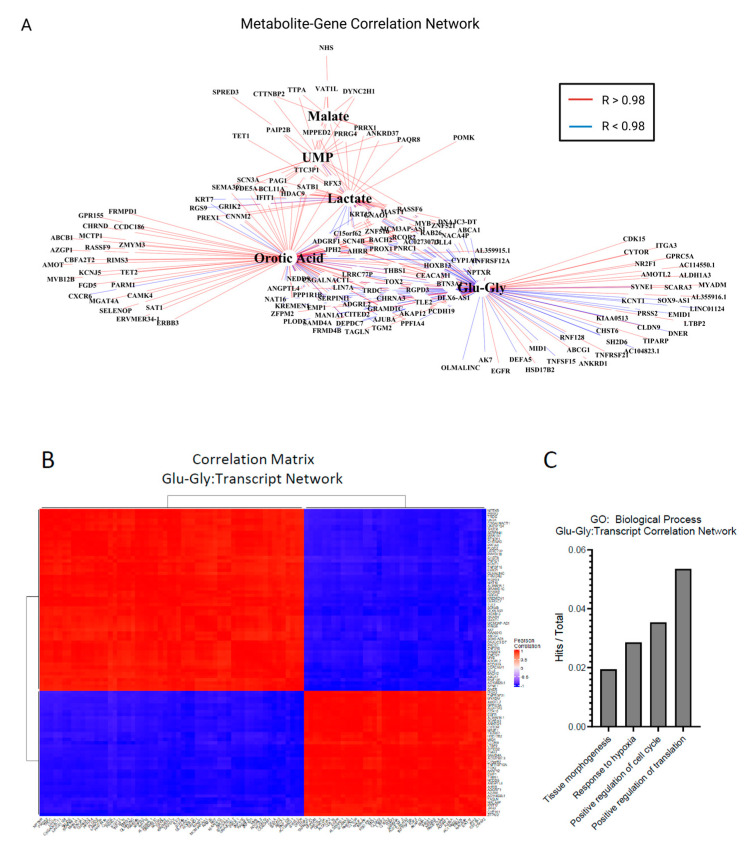
Multiomics pathway analysis of metabolic adaptation to hepatocytes. (**A**) Correlation network of differentially expressed genes (DEGs) and metabolites in SJH co-cultures compared to SJ control cultures. Gene names filtered from full bulk RNA-sequencing DEGs for significance of correlation to metabolites of interest (*p* < 0.001). Red lines indicate strong positive associations, and blue represent strong negative associations (R > |0.98|). (**B**) Hierarchical clustering of Pearson correlation matrix of transcripts highly correlated with glutamyl-glycine (Glu-Gly). (**C**) Gene counts with functional group membership of 98 transcripts were found to correlate strongly with glutamyl-glycine (Glu-Gly). Abbreviations: Glu-Gly, glutamyl-glycine dipeptide; UMP, uridine monophosphate.

Our 2D co-culture metabolomics pipeline and 3D transcriptional profiling both identified adaptations in amino acid, biosynthetic, and oxidative pathways. Therefore, we integrated these datasets in 2D to identify metabolite–gene relationships important to the SW480–hepatocyte microenvironment. Multiomics integration of a tumor cell transcription profile and metabolomic datasets can help establish the relationship between bulk metabolic adaptation and tumor cell phenotype. After bead isolation from hepatocytes and 3T3-J2s using antibodies against CD326 (EpCAM), RNA from SW480s was isolated and sequenced. Differentially expressed genes (adj. *p* < 0.01) were combined with significantly altered metabolite pools (Figure 3B) for univariate association analysis, recovering 627 mRNAs that were significantly correlated with 255 putative metabolites (*p* < 0.001, Supplementary Tables S3–S5). We further filtered this correlation matrix to only include very strong associations (R > |0.98|) to identify a subset of genes related to metabolic adaptation. A total of 151 unique genes correlated strongly with lactate, orotic acid, glutamyl-glycine (Glu-Gly), malate, and UMP (Figure 7A). In these analyses, positive correlations indicate co-expression in response to co-culture, while negative correlations indicate opposing expression patterns in response to co-culture. Given our findings of glutathione metabolism, we chose to look closer at the 98 genes that are associated with the metabolite glutamyl-glycine for gene–gene relationships (Figure 7B). GO enrichment analysis of biological processes of 98 genes associated with Glu-Gly revealed enrichment regulation of the cell cycle, response to hypoxia, and tissue morphogenesis (Figure 7C). Ontologies associated with all 151 genes further included adhesion, hypoxia, and angiogenesis (Supplementary Figure S6).

In this study, we have demonstrated a multiomics platform that uses LC-MS-based untargeted metabolomics approaches and bulk RNA-sequencing to reveal complex interactions in a mixed cell population. Additionally, these approaches can be translated to 3D microtissue organoid models. Bioinformatic interrogation of datasets suggests an important adaptation in the oxidative environment and in reactive oxygen species (ROS) homeostasis in the tumor microenvironment upon introduction to hepatocytes.

## 3. Discussion

This study employed a multiomics approach to uncover metabolic adaptation in the tumor–hepatocyte microenvironment using co-cultures of human colon adenocarcinoma cells (SW480) and primary rat hepatocytes, with the support of murine 3T3-J2 fibroblasts. By integrating untargeted metabolomics, ^13^C-stable isotope tracing untargeted metabolomics (ITUM), and transcriptomic analysis, we identified several critical metabolic alterations driven by the interaction between tumor cells and hepatocytes. First, co-culture of SW480s with primary hepatocytes significantly altered glucose metabolism. Hepatocytes reduced glucose consumption by 80% and lactate production by 94%, compared to SW480 monocultures. This suggests hepatocytes disrupt the classical Warburg effect in SW480 cells by influencing glucose metabolism [4,27,29]. Previous work indicates that aerobic glycolysis is an important adaptation for overcoming hypoxia within the liver; thus, suppression by hepatocytes may impact survival [11]. Hepatocytes display highly dynamic metabolism that serves to regulate glucose homeostasis. In the presence of high glucose levels, hepatocytes can direct excess glucose molecules to storage as glycogen, de novo lipogenesis, or to produce uridine [30]. Additionally, hepatocytes can use lactate for gluconeogenesis. Further study is necessary to deconvolute these intersectional and dynamic metabolic programs of varying glucose consumption (by both tumor cells and hepatocytes) and glucose production (hepatocytes) when cells are co-cultured in a manner that mimics the tumor microenvironment.

In response to reduced glucose consumption, SW480 cells adapted their fuel utilization. We observed a substantial reduction in ketone bodies and glutamine in the media of co-cultures, indicating increased consumption of these alternative substrates. In absence of glucose, tumor cells can reportedly shift to alternative fuels, such as glutamine; however, whether this plasticity extends to ketone bodies is not yet well understood. Ketogenesis has been a topic of interest in cancer research [31]. Studies have indicated a relationship between diminished expression of rate-limiting enzyme, 3-hydroxy-3-methylglutaryl synthase 2 (HMGCS2), and tumorigenesis [32,33,34]. Thus, it is also possible that diminished ketones in the media are due to reduced production through ketogenesis. Further, targeting ketogenesis has been proposed as a potential therapeutic strategy for inhibiting tumor progression, though responses in vivo vary between cancer types and stages [31,35,36,37,38]. Furthermore, other studies have indicated a relationship between metabolism, cancer progression, and response to therapeutic agents [39,40,41,42]. Our studies suggest an impact of SW480–hepatocyte interaction on ketone body metabolism may represent adaptation for tumor survival. As ketogenic capacity has also been shown to vary across the spectrum of metabolic dysfunction-associated steatotic liver disease (MASLD), further study of the relationship between ketogenesis, cancer, and liver health is warranted.

Untargeted metabolomics revealed additional changes in metabolites associated with the TCA cycle and energy metabolism. Notably, acetyl-CoA was elevated, while pyruvate, ATP, and NAD+ were decreased, highlighting key metabolic nodes affected by hepatocyte presence. The co-culture system revealed a possible metabolic exchange between hepatocytes and SW480 cells, particularly in purine and pyrimidine metabolism. SW480s in co-culture displayed enhanced salvage and reduced uric acid production, coupled with increased pyrimidine biosynthesis. Uric acid can regulate oxidative stress and activity of enzymes related to glucose metabolism, and it is associated with the development of metabolic syndrome [43]. Additionally, uric acid is linked with cancer risk [44,45]. Therefore, the modulation of uric acid levels and purine metabolism may be critical for targeting tumor growth. Further, due to comorbidities of cancer and obesity, the intersection of uric acid and metabolic syndrome in the tumor–hepatocyte niche may be an important area of further study. Recent work has established the pyrimidine intermediate, uridine, as an alternative fuel source for cancer cells in a glucose-restricted environment [46,47]. As uridine was elevated in the media of hepatocyte-containing cultures, it is possible SW480s utilize this metabolite to drive activity in the pyrimidine pathway. Further study is needed to understand how access to uridine impacts cancer metabolism in the liver and how flux through these nucleoside, and other biosynthetic, pathways may impact the oxidative environment, which could potentially affect emerging interventions targeting liver regeneration, such as portal vein embolization with stem cell augmentation (PVESA) [48].

^13^C-stable isotope tracing of mixed cell populations results in convoluted ^13^C-enrichment datasets that can be difficult to interpret for individual cell populations. For this reason, studies often employ conditioned media exchange to study intercellular metabolic dependencies. These studies are limited by the lack of cellular contact that may be relevant to metabolic interactions and the difficulty adapting these methods toward the more complex and advantageous preclinical models of the microenvironment, such as 3D microtissue organoids. Using unsupervised dimension reduction and association analyses, discriminant ^13^C-ITUM allows for pathway analysis by identifying (1) enriched isotopologues that discriminate groups and (2) metabolite–metabolite relationships within the discriminating isotopologue set. Therefore, discriminant ^13^C-ITUM is a form of pathway analysis that highlights co-enriched metabolic pathways characterizing mixed cell populations. This system-level approach enables specificity to substrate utilization with fewer constraints than metabolic flux analysis.

Transcriptomic profiling of SW480s revealed adaptation to metabolic pathways and enrichment of oncogenic pathways, including YAP, PTEN, and MYC. These signaling hubs have been implicated in the regulation of cancer cell metabolism [49,50,51]. Here, we observe their regulation in response to the presence of hepatocytes in the tumor microenvironment. Multiomics integration of metabolomics and RNA-seq data further showed that metabolic adaptations were associated with significant transcriptional changes in SW480 cells. These changes were linked to key biological processes, including adhesion, hypoxia response, and angiogenesis, indicating that tumor cells undergo functional adaptation in response to the hepatocyte microenvironment. These results are further supported by co-enrichment networks through a novel analysis of ITUM datasets that we have called discriminant ITUM. This analysis indicated the importance of glutathione synthesis in differentiating SW480–hepatocyte co-cultures from SW480 controls. Finally, these findings were supported by preliminary studies of a 3D microtissue organoid model, which demonstrated that co-culturing SW480 cells with hepatocytes induces similar transcriptional and metabolic changes observed in a 2D co-culture system.

While this study provides important insights into the metabolic interplay between tumor cells and hepatocytes, several limitations should be acknowledged. First, the use of 2D co-cultures, though effective in revealing key metabolic interactions, lacks the complexity of in vivo systems and may not fully capture the spatial and structural dynamics present in the liver microenvironment. Future work in advanced models such as organ-on-a-chip or 3D culture systems could provide more physiologically relevant insights. Additionally, our analysis primarily focused on the metabolic and transcriptional changes in tumor cells, leaving the metabolic impact on hepatocytes underexplored. Future work should involve a comprehensive assessment of hepatocyte responses to tumor interaction, including potential metabolic reprogramming. Additionally, utilization of our multiomics approach could further explore interactions between other tumor cell types and non-parenchymal cell populations residing in the liver, such as macrophages and cancer-associated fibroblasts [52,53,54,55]. Mechanistic studies targeting the identified metabolic nodes, such as altered glucose and ketone metabolism or nucleoside exchange, could further elucidate their roles in tumor progression and present potential therapeutic strategies to disrupt these metabolic dependencies.

## 4. Materials and Methods

### 4.1. Reagents

LCMS-grade water (H_2_O) (Fisher, W6-4), LCMS-grade methanol (MeOH) (Fisher Scientific, Waltham, MA, USA, A456-4), LCMS-grade acetonitrile (ACN) (Fisher Scientific, Waltham, MA, USA, A955-4), DMEM (high glucose) (Thermo Fisher Scientific, Waltham, MA, USA, 11965092), DMEM (no glucose) (Thermo Fisher Scientific, Waltham, MA, USA, A1443001), fetal bovine serum (Biotechne, Alpharetta, GA, USA, S11150), Pen/Strep (Thermo Fisher Scientific, Waltham, MA, USA, 15140122), L-glutamine (200 mM) (Thermo Fisher Scientific, USA, 25030081), Phosphate Buffered Saline (PBS) (no CaCl_2_ or MgCl_2_) (Thermo Fisher Scientific, Waltham, MA, USA, 14190144), Molecular Biology Grade Water (Corning, Corning, NY, USA, 46-000-CM), Pierce BCA Protein Assay Kit (Thermo Fisher Scientific, Waltham, MA, USA, 23225), Genomic DNA kit (blood and cultured cells) (IBI Scientific, Dubuque, IA, USA, IB47201). 0.25% Trypsin-EDTA (Corning 25-053-Cl), Hank’s Balanced Salt Solution (HBSS) (with CaCl_2_ or MgCl_2_) (Gibco, Waltham, MA, USA, 14025-076), Collagenase Type IV (Sigma Aldrich, Burlington, MA, USA, C5138), Human CD326 (EpCAM) MicroBeads (Miltenyi Biotec, San Diego, CA, USA, 130-061-101), CD16/CD32 Monoclonal Antibody (Invitrogen, Waltham, MA, USA, 14-0161-82), LS Magnetic Separation Columns (Miltenyi Biotec 130-042-401), QuadroMACS Magnetic Separator (Miltenyi Biotec 130-090-976), and RNeasy Mini Kit (Qiagen, Germantown, MD, USA, 74104).

### 4.2. Two- and Three-Dimensional Culture Platform

For both 2D and 3D cultures, 3T3-J2 fibroblasts (Kerafast, Boston, MA, USA, Catalog Number: EF3003) and SW480 cells (ATCC, lot Number: 700031955) were maintained in tissue culture flasks until ready for use. Primary rat hepatocytes (PRHs, XenoTech, Kansas City, MO, USA, Cryopreserved Male Wistar Rat Plateable Hepatocytes AMY 7 mil, Catalog Number: r3000.H15+ Lot No. 1210326) were thawed immediately before use. 3T3-J2 fibroblasts were growth-arrested using 1 µg/mL mitomycin-C for 4 h in culture before detachment using Trypsin-EDTA.

In 2D, 150k PRH, and 150k 3T3-J2 fibroblasts were plated per well for all HJ, SJH, and SJ wells. On day 7, 50k SW480 cells are seeded to SJH and SJ wells. On day 9, cell culture media were changed to exchange equimolar (22 mM) unlabeled glucose for non-radioactive, stable isotopically labeled 22 mM [U-^13^C_6_]glucose according to previously established protocols [18]. Cells and final conditioned media were collected on day 10.

Three-dimensional microtissue organoids were fabricated as previously described [25]. Briefly, plates were with 2% agarose and allowed to stiffen for 24 h. We then used a microwell stamp made from a PDMS mold to create 200 μM microwells that held the microtissues separately within the same well. After cleaning the microwells with a series of washes, we added media to the wells. We maintained the microtissues in Human Hepatocyte Maintenance Media (HHM) (43.25 mL 1x DMEM, 5 mL Bovine Serum, 750 μL HEPES, 1M, pH 7.6, 500 μL insulin/human transferring/selenous acid, and linoleic acid premix (Corning premix solution), 500 μL penicillin–streptomycin, 100X solution of 50 mg/mL stock, 0.5 μL dexamethasone, 10 mM in DMSO, 0.5 μL glucagon, and 0.7 mg/mL in 0.05 M acetic acid. During the study, we provided fresh media changes every 48 h by removing 300 μL from each well and replacing it with 300 μL of fresh media. We then removed the microtissues at their respective time point.

### 4.3. Quantification of Glucose and Lactate via 1 H NMR

First, ~50 µL of cell culture media was dried to completion in a SpeedVac (Fisher Scientific, Waltham, MA, USA) in the presence of D2O to aid in water suppression. Samples were reconstituted in D2O (99.9%) spiked with 0.3 mM d4-trimethyl-silyl propionate (TSP). 1H-NMR signals were acquired using a Bruker Avance III 600 NMR instrument equipped with a CryoProbe, and then the integrated intensities of the α-anomeric proton on glucose carbon-1, the methyl signal for lactate, and the tri-methyl signal from TSP were used to calculate molar concentrations of the respective substrates. For all 1H-NMR collections, spectra were collected by conventional pulse-and-collect measurements under quantitative conditions (10 ppm spectral range using ~15 μs [90°] excitation pulse and 22 s delay between each of 20 transients).

### 4.4. Untargeted Metabolomics and Isotope Tracing Untargeted Metabolomics Pipeline

Cells were harvested, metabolites extracted, and raw data acquired using liquid chromatography (LC) on a Thermo Vanquish UHPLC system, and full-scan high-resolution mass spectrometry (HR-MS) on a Thermo QExactive (Fisher Scientific) plus hybrid quadrupole-orbitrap mass spectrometer fitted with heated electrospray ionization source and operated in negative- and positive-polarity mode.

#### 4.4.1. Cell Collection for Metabolomics

Media were collected and snap-frozen, and then adherent cells were washed twice with 1 mL warm (37 °C) PBS (no MgCl_2_ or CaCl_2_), once with warm (37 °C) cell-culture-grade H_2_O, and then the entire plate was submerged into liquid nitrogen to snap-freeze cells, thus rapidly quenching metabolism. To preserve the metabolome, cells were scraped in 500 μL of cold (−20 °C) LCMS-grade MeOH per well of cells. Two wells were combined to form each replicate, n = 3 per group. Finally, MeOH was evaporated using a SpeedVac. Dried cell pellets were stored at −80 °C until analysis.

#### 4.4.2. Metabolite Extraction

Metabolites from cell pellets and conditioned media were extracted and analyzed by LC-MS untargeted metabolomics according to previously published protocols [18,56]. Briefly, cell pellets were reconstituted in 1 mL 2:2:1 (*v*/*v*/*v*) ACN/MeOH/water, then vortexed (30 s), flash-frozen in liquid nitrogen (1 min), and sonicated (25 °C, 10 min) in three cycles. After 1 h at −20 °C, samples were centrifuged at 15 k × g at 4 °C for 10 min. Supernatant was transferred to a fresh tube and dried by SpeedVac overnight, while the remaining cell pellet was stored at −80 °C after removing any remaining solvent for DNA quantification. Cell extracts are reconstituted in 40 µL 1:1 (*v*/*v*) ACN/water for analysis. Then, 20 µL of conditioned media was extracted in 80 µL 1:1 (*v*/*v*) ACN:MeOH. Media underwent a single cycle of vortex and sonication, and then followed the same procedure as cell pellets. Media samples were reconstituted in 200 µL 1:1 (*v*/*v*) ACN/water for analysis.

#### 4.4.3. Data Acquisition

For this study, polar metabolites were acquired using hydrophilic interaction chromatography (HILIC), and energy nucleotides were acquired via reverse phase (RP), using two unique UHPLC methods: [a] SeQuant ZIC-pHILIC column (2.1 × 150 mm, 5 μm) (Millipore Sigma, Burlington, MA, USA, 1.50460). Mobile phase A (MPA) was 95% H_2_O, 5% ACN, 10 mM ammonium acetate, and 10 mM ammonium hydroxide. Mobile phase B (MPB) was 100% ACN. The total run time was 50 min, flow rate was 2 mL/min, column chamber was set to 45 °C, and 2 µL sample was injected. Mobile-phase gradient was as follows: 0–0.5 min, 90% MPB; 0.5–30 min, 90–30% MPB; 30–31 min, 30% MPB; 31–32 min, 30–0% MPB; 32–33 min, 0–90% MPB; and 33–50 min, 90% MPB. [b] Energy nucleotides (ATP, ADP, and AMP) and redox nucleotides (NAD+ and NADH) were measured as previously described, with modifications. Briefly, metabolites were extracted from cells for ITUM in MeOH:ACN:H_2_O (2:2:1), and then extracts were separated and detected using ion-pairing RP UHPLC-MS/MS on a C18 column (Waters, Milford, MA, USA, Xbridge, 150 × 2.1mm, 3 μm). Nucleotides were detected as adducts of dibutylamine acetate on a Thermo QExactive Plus mass spectrometer, operated in positive ionization mode, using parallel reaction monitoring transitions as previously described.

For all metabolomics pipelines, both blanks and pooled quality control (QC) samples were injected periodically throughout the run. Blanks were ACN:H_2_O (1:1), and the QC sample was a pooled sample, including all naturally occurring and ^13^C-labeled samples. To aid in chemical feature identification, two additional samples were also injected. The first was a standard mix consisting of authentic standards for all expected analytes. The second was a pooled sample consisting only of the naturally occurring samples, analyzed via data-dependent analysis (DDA)–tandem mass spectrometry (MS/MS), using IE omics script and R-Studio [57].

#### 4.4.4. Data Preparation

Data processing and initial analysis were performed using Thermo Compound Discoverer 3.3. After raw mass spectra were uploaded, background ions were removed, retention times (RTs) for detected signals were aligned across samples, and chemical formulas were predicted. Then, grouped chemical features were profiled to determine compound identity, based on the following: (1) the *m*/*z* predicted from the chemical formula, (2) the RT compared to an authentic external standard, and (3) the MS/MS fragmentation pattern, compared to in-house standards or online databases.

For ITUM experiments, putatively identified metabolites and lipids were then carried forward and subjected to [^13^C] stable-isotope enrichment with correction for natural abundance. To carry out [^13^C] stable isotope tracing, all [^13^C] mass isotopomers (i.e., isotopologues) within the isotopic envelope of each identified metabolic pool were identified based on the diagnostic shift in *m*/*z* (Δ*m*/*z* = 1.0033 Da, natural abundance, 1.11% of all carbon) induced by the presence of ^13^C-labeled compounds. Raw ion counts for each isotopologue were extracted, summed, and expressed as a percentage of the total pool. After natural abundance correction, the fractional intensities were then graphed as a function of [^13^C] content, generating mass isotopologue distributions (MIDs) for each detected metabolite or lipid.

For static pool analysis, total ion counts were exported from Compound Discoverer and normalized to biomass (either total DNA or total protein). DNA was quantified from cell pellets after metabolite extraction using an IBI Scientific genomic DNA kit for cultured cells as previously described for metabolite normalization, following kit instructions [58]. Total protein was quantified using the Pierce BCA Protein Assay Kit from separately cultured and harvested samples in parallel with metabolomics experiments.

### 4.5. Quantification of Total Ketone Bodies

Acetoacetate (AcAc) and β-hydroxybutyrate (BOHB) were formally quantified using UHPLC-MS/MS as described previously [59,60]. Briefly, [U-^13^C_4_]AcAc and [3,4,4,4-D_4_]βOHB internal standards were spiked into ice-cold MeOH:ACN (1:1). Then, ketones were extracted, separated via reverse-phase UHPLC, and detected via parallel reaction monitoring (PRM) on a QExactive Plus hybrid quadrupole-orbitrap mass spectrometer.

### 4.6. Bulk RNA Sequencing and Analysis

For the 2D platform, cells were collected from the plates using 0.25% Trypsin-EDTA. For the 3D platform, microtissues were collected from the wells and dissociated by incubating at 37 °C with 0.1 mg/mL Collagenase Type IV in HBSS (+CaCl_2_, +MgCl_2_) for 30 min, then manually disrupted to form a single-cell suspension. For both platforms, 8 wells were combined to form replicates, n = 3 per group. The single-cell suspensions were incubated with MicroBeads against human CD326 (EpCAM) and antibody against CD16/CD32 to block nonspecific binding, and then they were isolated across Miltenyi LS magnetic separation columns. RNA was isolated from the sorted SW480 cells using a Qiagen RNeasy Mini Kit.

#### Bulk RNA-Seq Analysis

RNA-seq analysis was performed at the Minnesota Supercomputing Institute at the University of Minnesota. Briefly, Fastq files were first processed with the CHURP pipeline (version 0.2.3) [61] to perform adaptor trimming using trimmomatic (version 0.33) [62]; reads were then mapped to Homo sapiens GRCh38 genome using HiSat2 (version 2.1.0) [63]. Subread count was generated using the Subreads featureCounts tool (version 1.6.2) [64], using the Homo_sapiens.GRCh38.100.gtf annotation. Count data were filtered by removing genes that were less than 300 nt in length and including only genes that had a cpm (counts per million) value greater than 1 cpm in at least two sample replicates. The quasi-likelihood test was used to evaluate differential expression (DE) with edgeR (version 3.38.1) [65,66]. The Benjamini–Hochberg method was used to adjust *p*-values for multiple hypothesis testing and an adjusted *p*-value ≤ 0.05, and an absolute log_2_ fold change (|log_2_FC|) > 0 was used as a DE significance threshold. For gene ontology (GO pathway and GSEA analysis), the R package clusterProfiler (version 4.4.4) [67,68] was used. Normalized Enrichment Score (NES) indicates the distribution of genes across a ranked list and normalizes the correlation between gene sets and datasets to gene-set size, allowing for comparison. For GSEA analysis specifically, Hallmark and 18 selected C2 pathways were combined and used for testing (see full GSEA pathway results and selected C2 pathways in Supplementary Tables S1 and S2). Analysis of GSEA C6 oncogenic pathways was performed, and read counts were converted to normalized counts using the DESeq2 (version 1.42.0) R package and were further filtered to remove genes with zero expression across all samples. GSEA was then performed using the standalone software (version 4.3.2) developed by the Broad Institute, and statistical significance was assessed via gene-set permutation testing (1000 permutations) [69,70]. The GEO accession number is GSE282081.

### 4.7. Statistics and Multiomics Analysis

Descriptive data are expressed as mean and standard error (SEM) for continuous measures. Comparisons of metabolite abundance were made between HJ and SJH or SJ and SJH by unpaired *t*-test and corrected for multiple comparisons by Benjamini–Hochberg, using GraphPrism v10.2.3. Multiomics integration of bulk transcriptomic and metabolomics datasets were performed in R v4.2.3 using rcorr() from the Hmisc package (v.5.1) and the igraph package (v2.0.3) for visualization of correlations. Given the small da5.taset, only highly significant correlatons (*p*-value of Pearson correlation coefficient < 0.001) were included in downstream pathway and gene ontology analysis. Genes that correlated strongly with metabolites of interest were included as a set of gene IDs and fold changes in an ExpressAnalyst query for functional analysis [71].

Discriminant ITUM was performed using a curated dataset of positively identified metabolites by commercial standard and/or MS/MS match in mzCloud. Selected metabolites showed significant total enrichment in the SJH group after correction for natural abundance of ^13^C (1.11% of all carbon in nature), performed within the Compound Discoverer “stable isotope labeling” node based on predicted chemical formula. All isotopologues with enrichment were included for a given metabolite as individual variables, i.e., S_M0, S_M1, S_M2, and S_M3, included as four unique variables representing serine enrichment. Data are presented in 0–100 range and represent the percentage of the total metabolite pool detected by LC-MS in full scan (MS1). Total pools were calculated by sum of ion counts for each possible isotopologue (i.e., total ion counts of serine = S_M0 + S_M1 + S_M2 + S_M3; % enrichment of S_M3 = ion counts of S_M3/total ion counts of serine * 100). The resulting multivariate dataset was used for principal components analysis (PCA) to identify co-enriched isotopologues that discriminate SJH co-cultures from SJ or HJ controls. PCA was performed in GraphPrism v10.2.3. Data were standardized prior to PCA. A correlation network was graphed from the top, contributing isotopologues and distinguishing SJH from SJ controls, using the igraph package in the R environment for qualitative pathway analysis of glucose utilization in mixed cell populations. Joint-pathway analysis of 3D microtissue transcriptomics and metabolomics datasets was performed in MetaboAnalyst 4.0 [72].

## Figures and Tables

**Table 1:** Differential abundance of select metabolites in SJH co-cultures relative to HJ or SJ controls. Up arrow indicates increased abundance relative to the indicated control, HJ or SJ, while down arrow indicates decreased. FC, fold change; ctrl, control; HJ, hepatocyte+3T3-J2 co-culture; SJH, SW480+3T3-J2+hepatocyte co-culture; SJ, SW480+3T3-J2 co-culture; CoA, coenzyme A; NAD, nicotinamide adenine dinucleotide; ATP, adenosine triphosphate.

Putative Metabolite	Log_2_ (FC: SJH/ctrl)	Adjusted *p*-Value	Direction Relative to [HJ] or [SJ]
S-adenosylhomocysteine	1.87	0.0004	↑; HJ
Pyruvate	−1.05	0.0001	↓; HJ
Acetyl-CoA	−1.98	0.002	↓; HJ
Propionyl-CoA	−1.89	0.012	↓; HJ
Propionyl-CoA	5.01	0.0004	↑; SJ
Glutamyl-glycine	2.47	0.00007	↑; SJ
Acetyl-CoA	1.63	0.016	↑; SJ
Pyruvate	−3.60	0.0006	↓; SJ
Lactate	−2.78	0.0012	↓; SJ
NAD+	−1.85	0.012	↓; SJ
ATP	−1.09	0.03	↓; SJ

**Table 2:** Differential abundance of select metabolites in SJH co-cultures relative to HJ or SJ controls. FC, fold change; ctrl, control; HJ, hepatocyte+3T3-J2 co-culture; SJH, SW480+3T3-J2+hepatocyte co-culture; 1T1, 1-to-1 ratio of SJ to HJ metabolite extractant. Up arrow indicates increased abundance relative to 1T1 mixture.

Putative Metabolite	Log_2_ (FC: SJH/HJ)	Adjusted *p*-Value	Direction Relative to 1T1
Glutamyl-glycine	2.59	0.0018	↑
Uracil	2.24	0.0044	↑
Uridine diphosphate	1.42	0.021	↑
Aspartate	1.17	0.0018	↑
Malate	1.03	0.0029	↑

**Table 3:** Fold change in extracellular and intracellular metabolite abundance after 24 h of incubation relative to starting media (t0). Adjusted *p*-value by *t*-test comparing T24 abundance to T0 abundance with BH correction for multiple testing. Up arrows indicate increased abundance relative to T0 while down indicate decreased. Abbreviations: FC, fold change; E, change to Extracellular pool size; I, change to Intracellular pool size; HJ, hepatocyte+3T3-J2 co-culture; SJH, SW480+3T3-J2+hepatocyte co-culture; UDP, uridine diphosphate; NC, no change.

	Log_2_ (FC: T24/T0) [adj. *p*-Value]		
Putative Metabolite (E/I)	HJ	SJH	Direction Relative to T0
Hypoxanthine (E)	−1.36 [<0.0001]	−2.62 [<0.0001]	↓; ↓; ↓
Uric acid (E)	−3.97 [<0.001]	−4.20 [<0.001]	↓; ↓; ↑
Inosine (I)	1.00 [0.002]	1.27 [0.003]	↑; ↑; NC
Uridine (E)	2.36 [<0.0001]	1.94 [<0.001]	↑; ↑; NC
Orotic acid (E)	4.27 [<0.00001]	4.19 [<0.001]	↑; ↑; ↑
Aspartate (I)	0.04 [0.87]	0.55 [0.02]	NC; ↑; NC
Carbamoyl aspartate (I)	−0.16 [0.19]	5.47 [0.036]	NC; ↑; NC
UDP (I)	0.32 [0.62]	1.44 [0.017]	NC; ↑; NC
Uridine (I)	1.13 [0.10]	3.23 [0.005]	NC; ↑; NC
Orotic acid (I)	0.09 [0.62]	3.48 [0.029]	NC; ↑; NC

## Data Availability

The raw mass spectrometry data supporting the conclusions of this article will be made available by the authors on request. RNA-seq datasets were deposited with GEO accession number GSE282081. Access via https://www.ncbi.nlm.nih.gov/geo/query/acc.cgi?acc=GSE282081 (accessed on 17 February 2025).

## Supplementary Materials

The following supporting information can be downloaded at https://www.mdpi.com/article/10.3390/ijms26051976/s1.

## Abbreviations

The following abbreviations are used in this manuscript:

UHPLC	ultra-high-pressure liquid chromatography
MS	mass spectrometry
MS/MS	tandem mass spectrometry (MS^2^)
HCC	hepatocellular carcinoma
*m*/*z*	mass-to-charge ratio
RT	retention time
ITUM	isotope tracing untargeted metabolomics
