# Supplementary material for: Deciphering Colorectal Cancer–Hepatocyte Interactions: A Multiomics Platform for Interrogation of Metabolic Crosstalk in the Liver–Tumor Microenvironment
Source: Int J Mol Sci. 2025 Feb 25;26(5):1976. doi: 10.3390/ijms26051976 (PMC11900982; doi:10.3390/ijms26051976)
Supplement: Supplementary file 1 [file ijms-26-01976-s001.zip › suppFigures_IJMS.pdf]

Figure S1

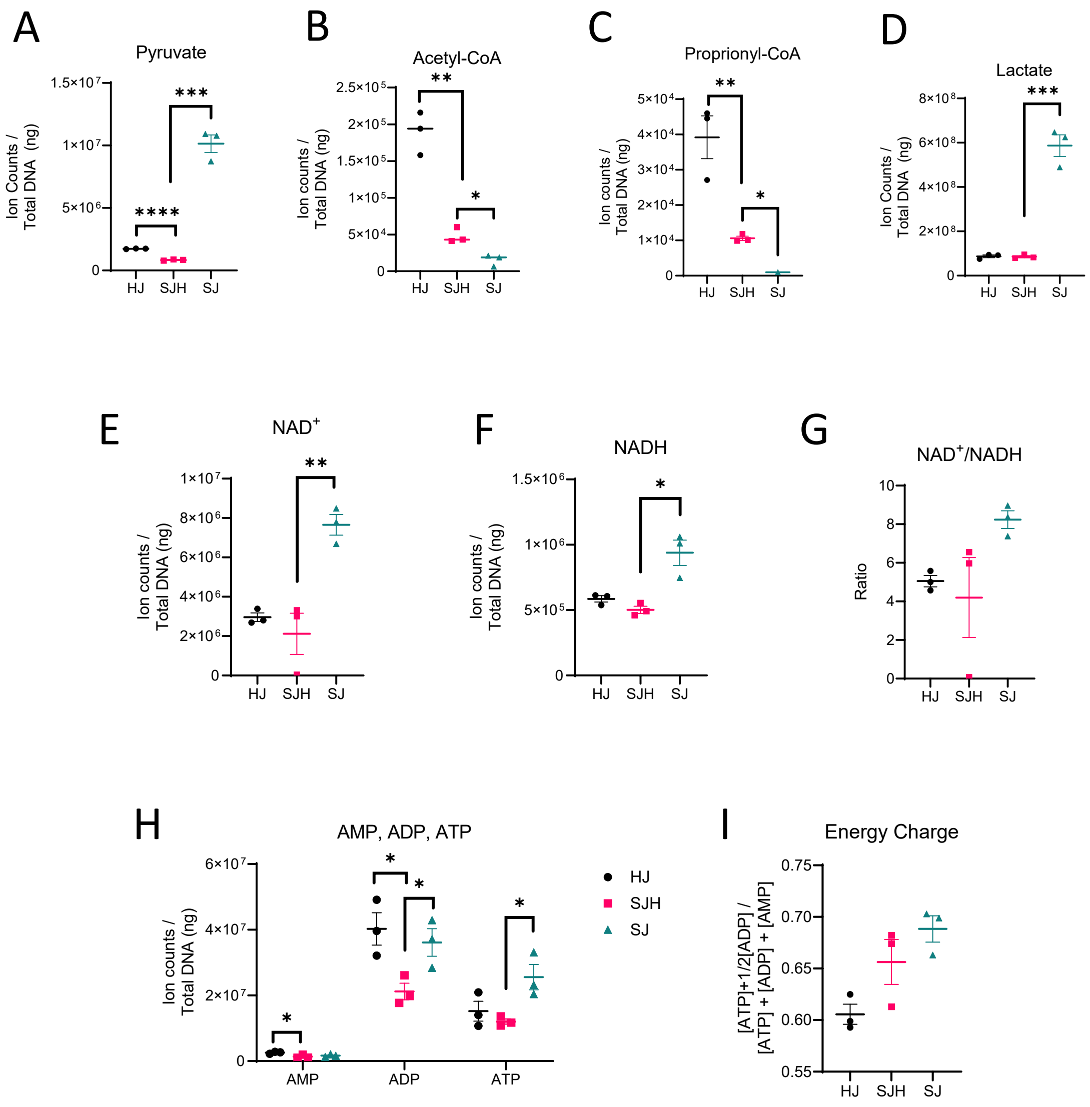

A
Pyruvate
Ion Counts / Total DNA (ng)
HJ SJH SJ
B
Acetyl-CoA
Ion counts / Total DNA (ng)
HJ SJH SJ
C
Proprionyl-CoA
Ion counts / Total DNA (ng)
HJ SJH SJ
D
Lactate
Ion Counts / Total DNA (ng)
HJ SJH SJ
E
NAD+
Ion counts / Total DNA (ng)
HJ SJH SJ
F
NADH
Ion counts / Total DNA (ng)
HJ SJH SJ
G
NAD+/NADH
Ratio
HJ SJH SJ
H
AMP, ADP, ATP
Ion counts / Total DNA (ng)
AMP ADP ATP
HJ
SJH
SJ
I
Energy Charge
[ATP]+1/2[ADP] / [ATP] + [ADP] + [AMP]
HJ SJH SJ

Figure S2

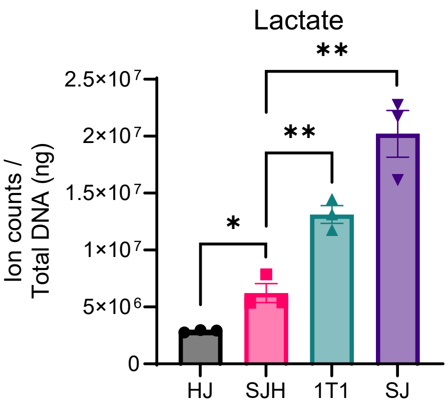

Lactate
Ion counts / Total DNA (ng)
2.5×10⁷
2×10⁷
1.5×10⁷
1×10⁷
5×10⁶
0
HJ
SJH
1T1
SJ
*
**
**

Figure S3

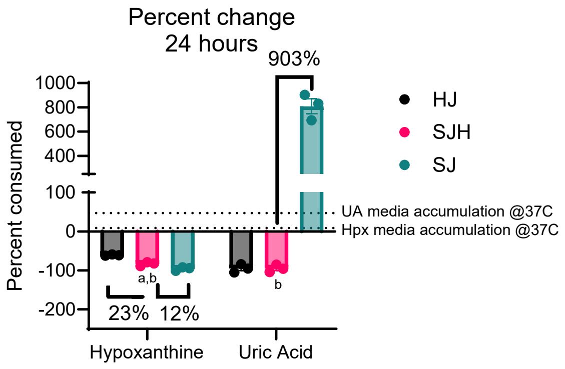
Percent change
24 hours
903%
1000
800
600
400
100
0
-100
-200
Percent consumed
UA media accumulation @37C
Hpx media accumulation @37C
a,b
b
23%
12%
Hypoxanthine
Uric Acid
HJ
SJH
SJ

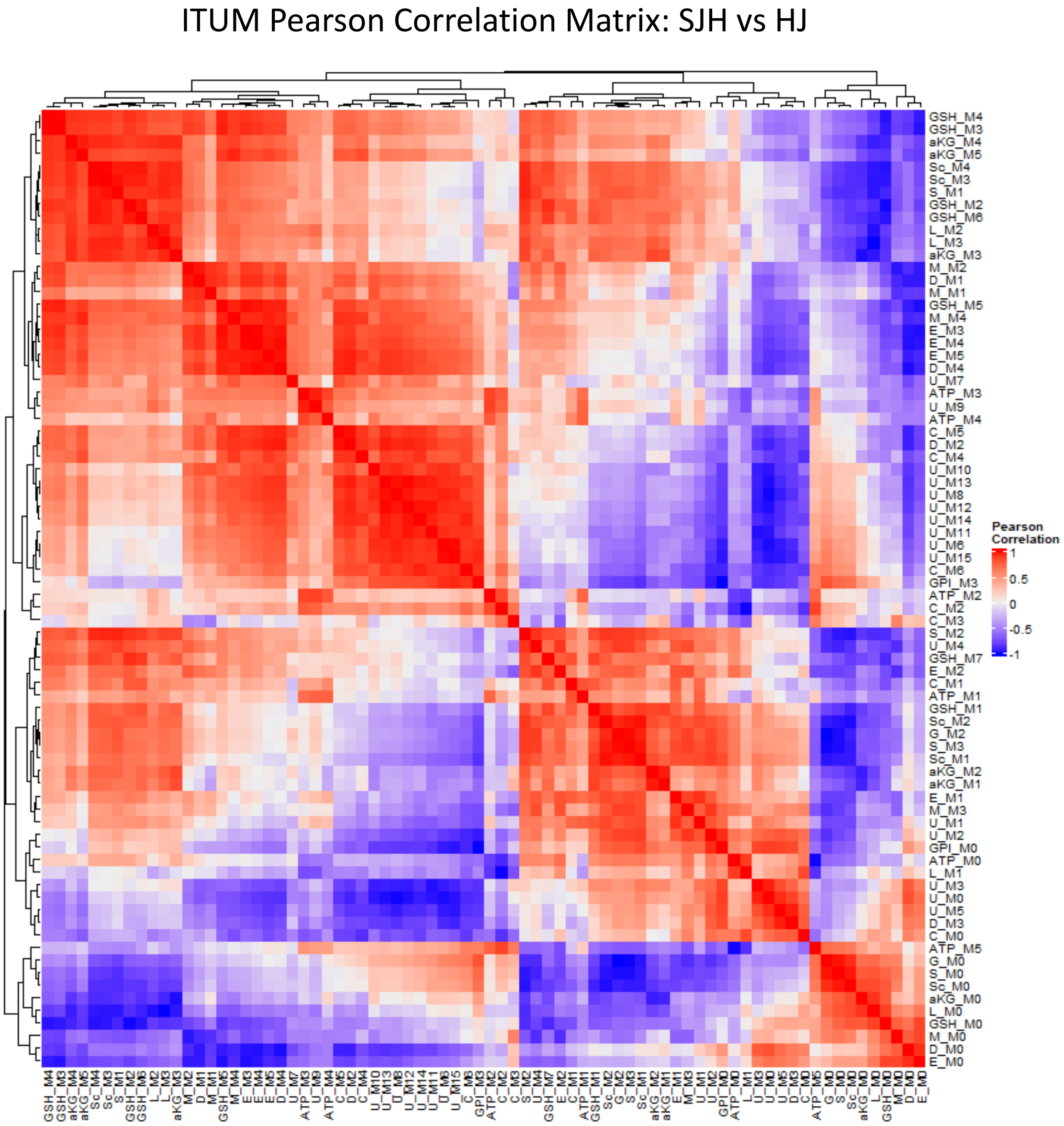
ITUM Pearson Correlation Matrix: SJH vs HJ
Pearson Correlation
1
0.5
0
-0.5
-1
GSH_M4
GSH_M3
aKG_M4
aKG_M5
Sc_M4
Sc_M3
S_M1
GSH_M2
GSH_M6
L_M2
L_M3
aKG_M3
M_M2
D_M1
M_M1
GSH_M5
M_M4
E_M3
E_M4
E_M5
D_M4
U_M7
ATP_M3
U_M9
ATP_M4
C_M5
D_M2
C_M4
U_M10
U_M13
U_M8
U_M12
U_M14
U_M11
U_M6
U_M15
C_M6
GPI_M3
ATP_M2
C_M2
C_M3
S_M2
U_M4
GSH_M7
E_M2
C_M1
ATP_M1
GSH_M1
Sc_M2
G_M2
S_M3
Sc_M1
aKG_M2
aKG_M1
E_M1
M_M3
U_M1
U_M2
GPI_M0
ATP_M0
L_M1
U_M3
U_M0
U_M5
D_M3
C_M0
ATP_M5
G_M0
S_M0
Sc_M0
aKG_M0
L_M0
GSH_M0
M_M0
D_M0
E_M0

Figure S5

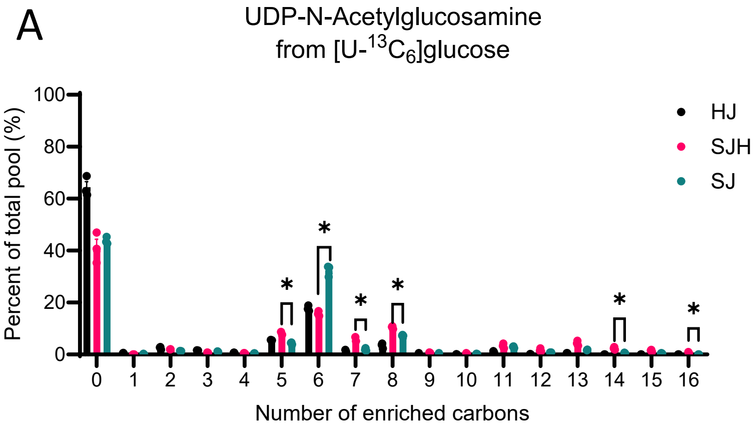
A
UDP-N-Acetylglucosamine
from [U-13C6]glucose
Percent of total pool (%)
100
80
60
40
20
0
0 1 2 3 4 5 6 7 8 9 10 11 12 13 14 15 16
Number of enriched carbons
HJ
SJH
SJ

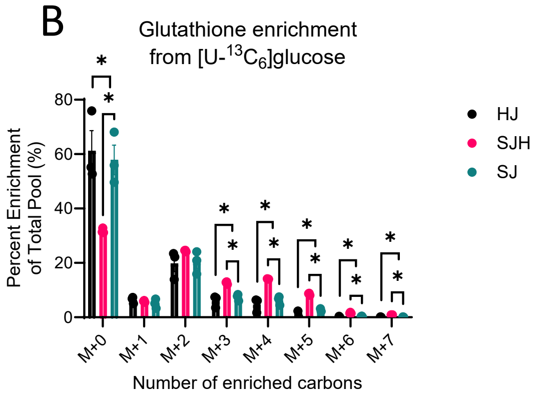
B
Glutathione enrichment
from [U-13C6]glucose
Percent Enrichment
of Total Pool (%)
80
60
40
20
0
M+0 M+1 M+2 M+3 M+4 M+5 M+6 M+7
Number of enriched carbons
HJ
SJH
SJ

Figure S6

A

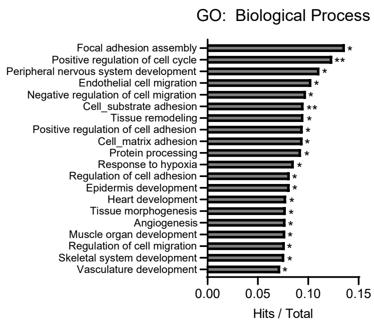
GO: Biological Process
Focal adhesion assembly *
Positive regulation of cell cycle **
Peripheral nervous system development *
Endothelial cell migration *
Negative regulation of cell migration *
Cell_substrate adhesion **
Tissue remodeling *
Positive regulation of cell adhesion *
Cell_matrix adhesion *
Protein processing *
Response to hypoxia *
Regulation of cell adhesion *
Epidermis development *
Heart development *
Tissue morphogenesis *
Angiogenesis *
Muscle organ development *
Regulation of cell migration *
Skeletal system development *
Vasculature development *
0.00
0.05
0.10
0.15
Hits / Total

B

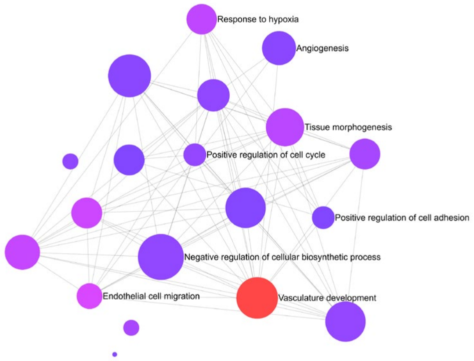
Response to hypoxia
Angiogenesis
Tissue morphogenesis
Positive regulation of cell cycle
Positive regulation of cell adhesion
Negative regulation of cellular biosynthetic process
Endothelial cell migration
Vasculature development

Figure S1. Metabolite abundance in 2D co-cultures. Ion counts after normalization to total ng DNA of: (A) pyruvate, (B) Acetyl-CoA, (C) Propionyl-CoA, (D) Lactate, (E) NAD+, (F) NADH, and (H) AMP, ADP, ATP nucleotides. (G) Ratio of NAD+ to NADH ion counts. (I) Calculated energy charge of each co-culture group based on ion counts of AMP, ADP, and ATP. Significance tested using unpaired t-test, comparison HJ vs SJH or SJ vs SJH, and corrected for multiple comparisons using Benjamini-Hochberg method. *: p adj. < 0.05, **: p adj. < 0.01, ***: p adj. < 0.001, ****: p adj. < 0.0001.

Figure S2. Lactate abundance in co-culture and 1T1 dilution. Bar graph of total ion counts after normalization to DNA compared to analytical dilution (1T1). Significance tested using unpaired t-test, comparison HJ vs SJH or SJ vs SJH, and 1T1 vs SJH; corrected for multiple comparisons using Benjamini-Hochberg method. *: p adj. < 0.05, **: p adj. < 0.01

Figure S3. Percent change in media purines. Percent difference in media after 24h incubation with co-cultured cells where time point 0 represents starting media abundance prior to cell exposure. Dotted lines represent accumulation of hypoxanthine (Hpx) and uric acid (UA) after 24h at 37oC in media in absence of cells. Statistical comparison by unpaired t-test; letters indicate significance in comparison to HJ controls (“a”) or SJ controls (“b”).

Figure S4. Correlation matrix of SJH vs HJ ITUM in 2D co-cultures. Correlation matrix assessing co-enriched isotopologues in response to presence of hepatocytes (SJ, SJH cultures). Red gradient represents positive associations while blue represents negative associations. Correlations measuring by Pearson correlation method.

Figure S5. Distribution of 13C enrichment. Percent enrichment of total pools of (A) Uridine diphosphate N-acetyglucosamine and (B) glutathione after 24h incubation with [U-13C6]glucose at 37oC. *: p adj. < 0.05

Figure S6. Multiomic pathway analysis. (A) Gene counts with functional group membership of 151 genes found to correlate strongly with glutamyl-glycine (Glu-Gly), orotic acid, lactate, uridine monophosphate, and malate. (B) Functional network of shared genes in represented transcriptional profile with strong metabolite-gene associations; analysis

performed using ExpressAnalyst. (C) Scatter plot of joint pathway analysis from MetaboAnalyst v4.0 using DEGs and full static metabolomics dataset based on associated fold changes. X and y axes show enrichment score in genes and metabolite peaks, respectively.
